# Aging in Single-Room Occupancy Hotels: Using Photovoice to Co-Create Knowledge With Older Adults

**DOI:** 10.1093/geroni/igaf122.1062

**Published:** 2025-12-31

**Authors:** Jarmin Yeh, Kattia Suarez Vargas, Howard Thornton, Susi Stadler

**Affiliations:** University of California, San Francisco, San Francisco, California, United States; University of California, San Francisco, San Francisco, California, United States; At Home With Growing Older, Berkeley, California, United States; At Home With Growing Older, Berkeley, California, United States

## Abstract

Single-room occupancy hotels (SROs) in San Francisco’s Tenderloin provide crucial affordable housing for low-income, formerly unhoused, and marginalized populations. As residents age, these buildings are transforming into naturally occurring retirement communities, posing complex challenges for aging in place. Despite their important role, SROs frequently have neglected maintenance, deteriorating conditions, and safety concerns, and are often located in neighborhoods with higher rates of noise, crime, and substance use. While research has explored characteristics of older SRO residents, their experiences of aging in place remain understudied. This qualitative project used participatory methods to amplify the contributions of SRO residents. We formed an Advisory Council of SRO residents with lived expertise and established a partnership with two community stakeholders: a nonprofit specializing in home adaptations and a senior center. Through human-centered design activities and culturally responsive bilingual facilitation, the Advisory Council examined intersecting impacts of ageism and racism across socioecological levels, leading to a co-created action plan for future collaborative projects. Our subsequent photovoice study engaged twelve diverse participants in documenting their environments with cameras, producing rich visual and narrative data that highlighted the multifaceted social, spatial, cultural, and temporal dynamics of daily life in and around SROs. This approach revealed both resistance and vulnerabilities among residents while assessing systems of oppression from internalized to structural levels. By prioritizing the perspectives of marginalized older adults, this project fostered community empowerment and enhanced capacity for civic engagement. Findings offer insights for co-developing resident-centered approaches to support aging in place within these vital housing communities.

